# Wandering Thoughts

**Published:** 1857-06

**Authors:** H. M. Sheerar

**Affiliations:** Wellsville, N. Y.


### WANDERING THOUGHTS.

MESSRS. EDITORS :—As you have invited any of the profession who have a stray idea, to communicate to you, I venture a few wandering thoughts.

I have had some difficulty in extracting the inferior molars, when the crowns are decayed down to the gums, so I ordered a forceps made by Jones, White & Co., which proves to be a good thing, and quite effectual in such cases. They are made in such a manner that the closing of the beaks will many times force the tooth out.

The general shape of the instrument is similar to a right inferior molar forceps. The beaks are *round*, terminating in a blunt point, which will pass between the fangs, and almost always cause the tooth to fly out of the patient's mouth. I consider it very valuable, and would as soon part with almost any other instrument.

In making my casts, I do not wind paper or sheet lead around the impression, but have made some tin rings (not exactly rings, because not round,) of different sizes, to correspond with my different sized wax holders or cups; after the impression is taken, I select the proper sized "ring," press it into the outer edge of the impression; fill up with the plaster, and when hard, remove; a slight jar will cause the model to fall from the ring, which will be very smooth and well proportioned, needing but little trimming to be ready for moulding.

I have fixed a pair of pliers for splitting rivets of the teeth after being lined. I simply procured a pair of shoemakers' punch pliers that had been thrown aside, and fitted a little chisel where the punch was; placing the tooth in the pliers, I can split the rivet with ease.

I should like to ask you, Messrs. Editors, or any one else, how you would manage to fill a lower second molar tooth, the cavity being on the back side, or as Harris has it, "the posterior approximal surface," *without having the cavity get wet.*

Supposing the patient's mouth is rather small, and begins to close on your fingers, as soon as you begin to operate. *I* find considerable trouble in plugging such teeth. Do others?

WELLSVILLE, N. Y., 1857. H. M. SHEERAR.

